# Cardiovascular toxicity risk assessment of tyrosine kinase inhibitors: a pharmacovigilance study using the VigiBase database

**DOI:** 10.3389/fphar.2024.1472008

**Published:** 2024-12-02

**Authors:** Yusuke Igawa, Hirofumi Hamano, Satoru Esumi, Tatsuaki Takeda, Makoto Kajizono, Ryo Kikuoka, Ikuya Kimura, Yoshito Zamami

**Affiliations:** ^1^ Department of Clinical Pharmacology and Pharmacy, Okayama University, Okayama, Japan; ^2^ Department of Pharmacy, Okayama University Hospital, Okayama, Japan; ^3^ Department of Clinical Drug Evaluation, Faculty of Pharmaceutical Sciences, Kobe Gakuin University, Kobe, Japan; ^4^ Department of Education and Research Center for Clinical Pharmacy, Faculty of Pharmaceutical Sciences, Okayama University, Okayama, Japan; ^5^ Department of Pharmacy, Okayama City Hospital, Okayama, Japan

**Keywords:** tyrosine kinase inhibitors, cardiotoxicity, VigiBase, database, cancer

## Abstract

**Introduction:**

Advances in the early detection and treatment of cancer have significantly improved the prognosis of patients with cancer. Tyrosine kinase inhibitors (TKIs) are effective targeted treatments for various malignancies that act by inhibiting kinase activity. Although these drugs share a common mechanism of action, they differ in their targeted kinases, pharmacokinetics, and side effects. TKIs can cause cardiovascular side effects, which adversely affect the prognosis of cancer survivors. This study aimed to assess the risk of cardiac toxicity associated with TKIs using the World Health Organization Global Database, VigiBase.

**Methods:**

We conducted a cross-sectional analysis of data from VigiBase, a comprehensive global database of suspected drug reactions. The dataset included reports up to December 2022. We identified patients treated with Food and Drug Administration-approved TKIs and analyzed their age and sex data. The primary outcome was cardiovascular impairment, defined by 21 preferred terms in the Medical Dictionary for Regulatory Activities Terminology version 25.1. Disproportionality analysis using the reported odds ratio was performed to detect adverse cardiovascular signals. Statistical analyses were conducted using R 3.3.2, with a P-value <0.05 considered significant.

**Results:**

Of the 32, 520, 983 reports in VigiBase, 23, 181, 539 were eligible for the analysis. Significant cardiovascular signals were identified for 17 TKIs, including erlotinib, gefitinib, and imatinib. Stratified analyses revealed potential sex- and age-related differences in the risk of adverse events. Heatmaps indicated significant signals for drugs such as lapatinib in males and gefitinib in younger patients.

**Discussion:**

Our findings indicate that some TKIs, particularly those classified as VEGFR, BCR-ABL, and BTK, pose similar risks of cardiotoxicity, while others, including EGFR, HER2, and ALK TKIs, exhibit varied risk profiles. These results underscore the importance of individualized risk assessment and management of TKI-treated patients. In conclusion, this study provides valuable insights into the cardiotoxic risk of TKIs, which is essential for developing tailored treatment plans.

## 1 Introduction

Advances in the early detection and treatment of cancer have greatly improved the prognosis of patients with cancer. Tyrosine kinase inhibitors (TKIs) are particularly effective for the targeted treatment of various malignancies (Pottier et al., 2020). They have in common a significantly high affinity for the adenosine triphosphate (ATP)-binding pocket of tyrosine kinase (TK) and act by inhibiting the transfer of a phosphate group from ATP to a tyrosine residue, resulting in inhibits TK activity (Jiao et al., 2018). Imatinib was first introduced in cancer therapy, and many other agents are currently in use, including gefitinib, erlotinib, sorafenib, sunitinib, and dasatinib. Although these drugs have the same mechanism of action of inhibiting TKs, they differ in their spectrum of targeted kinases, pharmacokinetics, and substance-specific side effects (Hartmann et al., 2009). TKIs inhibit TK in cancer and non-cancer cells, and their effects on normal tissues cause side effects. TKI-based cancer treatment requires long-term drug therapy, and the development of cardiovascular disease due to prolonged TKI use adversely affects the prognosis of “cancer survivors” (Boskabadi et al., 2023).

Cardiotoxicity associated with congestive heart failure has been reported in patients treated with imatinib and sunitinib (Chu et al., 2007; Turrisi et al., 2010). The cardiotoxicity of TKIs ranges from asymptomatic QT prolongation to decreased left ventricular ejection fraction, symptomatic congestive heart failure, acute coronary syndrome, and myocardial infarction (Binzaid et al., 2021; Liu et al., 2023; Orphanos et al., 2009). Not all TKIs have the same toxicity in the myocardium. However, it remains unclear whether it is appropriate to discuss the cardiotoxicity of TKIs as a class-specific issue or on a drug-by-drug basis. Because cardiotoxicity has a low incidence of side effects, its detection is not typically included in most clinical trials, resulting in an unknown incidence of cardiotoxicity (Jin et al., 2020). Furthermore, clinical differences in the incidence of cardiotoxicity between drugs have not been adequately investigated (Destere et al., 2024).

Although cardiac toxicity from TKIs is widely recognized, studying TKIs at a single institution is challenging because of the small treated patient population. Consequently, the risk of cardiac toxicity has not been adequately assessed, particularly for drugs that have not yet been approved. Recently, attempts have been made to evaluate the potential risks of drugs that are challenging to analyze using conventional methods and large databases. For example, a study identified cardiovascular side effects of immune checkpoint inhibitors (ICIs) using the Food and Drug Administration Adverse Event Reporting System database (FAERS) and found that each ICI has a different side effect profile (Chen et al., 2021). In addition, a study has made an impressive attempt to characterize the side effect profile of ICIs using two approaches as follows: disproportionality analysis with FAERS and meta-analysis (Tyagi and Anoop, 2024). Many new signals associated with different organ classes were identified in the abovementioned study. Therefore, we considered using a large adverse event report database to analyze the cardiovascular adverse event profile of TKIs. This study aimed to assess the risk of cardiac toxicity from anticancer drugs using the World Health Organization’s global database of individual case reports known as VigiBase.

TKIs are known to have various off-target effects, and the mechanisms behind the development of cardiac toxicity vary significantly between different TKI types (Chen et al., 2008; Force and Kolaja, 2011; Shyam et al., 2023). Therefore, comparing cardiac events across different TKIs and evaluating their relative risks is crucial for identifying previously unrecognized cardiac events due to the lack of clinical data. Large-scale real-world data is essential for this purpose. VigiBase, being a global repository of individual case safety reports, provides a broader dataset than single-institution studies, allowing for a more extensive analysis of risk factors (Mitsuboshi et al., 2023; Nishiuchi et al., 2022). This study aimed to assess the cardiac toxicity risks associated with TKIs using VigiBase, contributing to safer treatment strategies for patients with cancer.

## 2 Materials and methods

### 2.1 Study design and setting

Our study used a cross-sectional design and analyzed data from VigiBase, the World Health Organization’s global database of suspected adverse events associated with medicinal products. VigiBase is one of the largest databases globally, containing more than 27 million reports collected from over 130 countries. It is a global repository of post-marketing safety reports, and pharmacovigilance using this database provides warnings about potential issues with marketed medications (Lindquist, 2008). Researchers can access VigiBase via the Uppsala Monitoring Centre (UMC) to facilitate replication and verification of the data used in this study. Access to the database requires formal collaboration with UMC, and further details about accessing VigiBase can be found on their official website: https://who-umc.org/vigibase/vigibase/.

### 2.2 Analysis of VigiBase data

**FIGURE 1:**
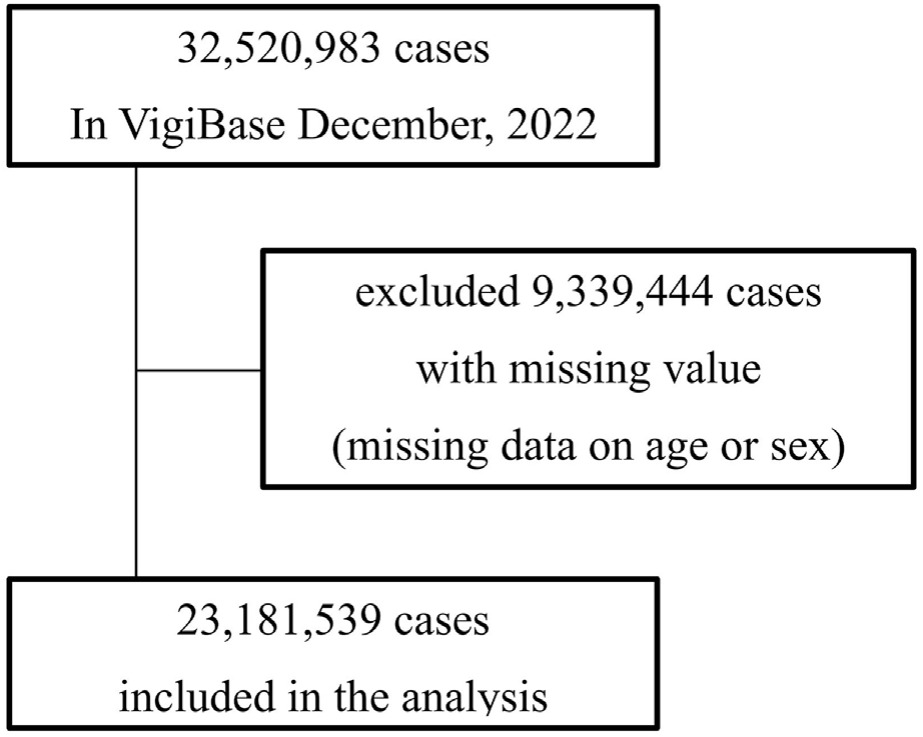
This flowchart illustrates the selection process of adverse event reports included in the analysis from VigiBase as of December 2022. Initially, 32, 520, 983 cases were reported. Of these, reports with missing data on age or sex totaling 9,339,444 cases were excluded from the analysis. The final dataset included 23, 181, 539 cases.

Data recorded from 1968 to December 2022, the maximum duration available, were analyzed. A flowchart illustrating the patient selection process is shown in Figure 1. The methodology, using statistical algorithms developed by the World Health Organization Collaborating Centre as described, underpins our continued efforts in exploring this dataset to identify medications at risk. Suspected duplicates are detected in VigiBase using the automatic algorithm vigiMatch (Norén et al., 2007). This approach has been instrumental in refining our analysis process, starting with the elimination of duplicate reports to ensure data integrity. For suspected duplicates, the report with the highest vigiGrade (Bergvall et al., 2014) completeness score is flagged.

Subsequently, patients who received TKIs were identified, and their age and sex data were included in the eligibility dataset. The TKIs investigated were approved by the Food and Drug Administration as of December 2022. They included BCR-ABL TKIs (asciminib, bosutinib, dasatinib, imatinib, nilotinib, and ponatinib), receptor-type TKIs (EGFR TKIs: afatinib, dacomitinib, erlotinib, gefitinib, icotinib, olmutinib, osimertinib, and rociletinib; HER2 TKIs: lapatinib, neratinib, and tucatinib; VEGFR TKIs: axitinib, cediranib, and tivozanib; and FGFR TKIs: erdafitinib, futibatinib, infigratinib, and pemigatinib), ALK inhibitors (alectinib, brigatinib, ceritinib, crizotinib, and lorlatinib), JAK inhibitors (fedratinib, pacritinib, and ruxolitinib), and BTK inhibitors (acalabrutinib, ibrutinib, and zanubrutinib). The number of side effect reports for each drug selected in this study is shown in Table 1.

**TABLE 1:** Number of reports analyzed by drug.

Drug name	Number of reports	Drug name	Number of reports
Acalabrutinib	1,362	Icotinib	207
Afatinib	9,539	Imatinib	39,207
Alectinib	2,630	Infigratinib	19
Asciminib	164	Lapatinib	11,005
Axitinib	10,655	Lolatinib	1,800
Bosutinib	4,742	Neratinib	469
Brigatinib	779	Nilotinib	16,465
Cediratinib	143	Olmutinib	372
Ceritinib	1,996	Osimertinib	8,115
Crizotinib	8,699	Pacritinib	39
Dacomitinib	325	Pemigatinib	46
Dasatinib	19,384	Ponatinib	4,245
Erdafitinib	240	Rociletinib	12
Erlotinib	20,030	Ruxolitinib	16,025
Fedratinib	290	Tivozanib	164
Futibatinib	9	Tucatinib	666
Gefitinib	7,571	Zanubrutinib	292
Ibrutinib	29,650		

We focused on cardiovascular impairment as the primary outcome, defined using 21 preferred terms from the Medical Dictionary for Regulatory Activities Terminology version 25.1. This definition aligns with cardiovascular adverse events as interpreted in a previous study (Niimura et al., 2023). The adverse events were categorized as suspected, interacting, or concomitant. All patients were included in the analysis, with age and sex used as covariates (Table 2).

**TABLE 2:** Definition of outcome.

Adverse event groups	MedDRA terms
Hypertension	Hypertension (SMQ narrow 25.1)
Arterial embolism and thrombosis	Embolic and thrombotic events, arterial (SMQ narrow 25.1)
Myocardial infarction	Myocardial infarction (SMQ narrow 25.1)
Central nervous system ischemia	Ischemic central nervous system vascular conditions (SMQ narrow 25.1)
Bradyarrhythmias	Bradyarrhythmia terms, nonspecific (SMQ narrow 25.1) Conduction defects (SMQ narrow 25.1) Disorders of sinus node function (SMQ narrow 25.1)
Endocardial disorders	Endocardial disorders (HLGT 25.1)
Supraventricular tachyarrhythmias	Supraventricular tachyarrhythmias (SMQ narrow 25.1)
Ventricular tachyarrhythmias	Ventricular tachyarrhythmias (SMQ narrow 25.1)
Cardiomyopathy	Cardiomyopathy (SMQ narrow 25.1)
Respiratory failure	Respiratory failure (SMQ narrow 25.1)
Heart failure	Cardiac failure (SMQ narrow 25.1)
Shock	Shock-associated circulatory or cardiac conditions (excl torsade de pointes) (SMQ narrow 25.1)
Myocarditis	Non-infectious myocarditis (HLT 25.1)
Pericarditis	Non-infectious pericarditis (HLT 25.1) and pericardial disorders NEC (HLT 25.1)
Venous embolism and thrombosis	Embolic and thrombotic events, venous (SMQ narrow 25.1)
Hemorrhage (clinical events)	Hemorrhage terms (excl laboratory terms) (SMQ narrow 25.1)
Bleeding-related laboratory abnormalities	Hemorrhage laboratory terms (SMQ narrow 25.1)
Cerebral hemorrhage	Hemorrhagic central nervous system vascular conditions (SMQ narrow 25.1)
Pulmonary hypertension	Pulmonary hypertension (SMQ narrow 25.1)
Vasculitis	Vasculitis (SMQ narrow 25.1)
Temporal arteritis/Polymyalgia rheumatica	Polymyalgia rheumatica (PT 25.1) and Giant cell arteritis (PT 25.1)

### 2.3 Statistical analysis

First, a disproportionality analysis using the reported odds ratio (ROR) was performed to detect adverse cardiovascular signals caused by TKIs. Adverse event reports were categorized into four groups as follows: (a) number of reports of myocarditis with the TKI of interest, (b) number of reports of other adverse events with the TKI of interest, (c) number of reports of myocarditis without the TKI of interest, and (d) number of reports of other adverse events without the TKI of interest. The ROR was calculated as ROR = [a/(a + b)]/[c/(c + d)]. Furthermore, the RORs, 95% confidence intervals (CIs), and significance levels were calculated using Fisher’s exact test (Faillie, 2018).

Next, we used the Data Mining disproportionality analysis based on the information component (IC) for each drug from VigiBase. IC is a Bayesian indicator of disproportionate reporting that estimates the strength of the association between a drug and an adverse cardiovascular signal (Van Holle and Bauchau, 2014). IC025 is the lower limit of the 95% CIs for the IC. A positive IC025 value is the traditional threshold used in statistical signal detection. This allowed us to detect adverse cardiovascular signals that are significantly associated with over-reporting for each TKI. A signal detection threshold (IC025 > 0) was applied to the detected adverse event to improve selectivity and minimize the risk of examining unclear TKI–cardiovascular adverse event associations. Among the adverse events identified using this approach, the adverse cardiovascular signals were considered to reflect the adverse events of the TKI class.

Furthermore, we evaluated the impact of sex and age on the detection of adverse cardiovascular signals using RORs. RORs were calculated for male or female, older patients (≥65 years), or younger patients (<65 years) to assess whether an impact exists on signal detection.

All statistical analyses were performed using R 3.3.2 (R Foundation for Statistical Computing, Vienna, Austria). A P < 0.05 was considered statistically significant.

### 2.4 Creation of heatmaps

Heatmaps were created based on the calculated RORs and P-values, categorized as follows: ROR values >1 and <1 were red and green, respectively. The color intensity increased as the P-value decreased, with darker shades indicating smaller P-values. In this study, the two darkest shades of red, corresponding to P < 0.01, were defined as significant signals.

## 3 Results

### 3.1 Characteristics of the patient cohort

Of the 32, 520, 983 reports collected in VigiBase until December 2022, 23, 181, 539 were eligible for analysis after excluding 9,339,444 reports with missing age or sex data. Table 1 presents the characteristics of the included patients. A total of 14, 095, 678 and 9,085,861 reports involved female and male patients, respectively. Reports from patients aged <65 and ≥65 years amounted to 16, 800, 356 and 6,381,183, respectively (Table 3).

**TABLE 3:** Characteristics of patients.

Characteristics	Number of reports (%)	
Patient Sex		
Female	14,095,678	(60.8)
Male	9,085,861	(39.2)
Patient age		
<65 years old	16,800,356	(72.5)
(Younger patients)		
≥65 years old	6,381,183	(27.5)
(Older patients)		

### 3.2 Heatmap

**FIGURE 2:**
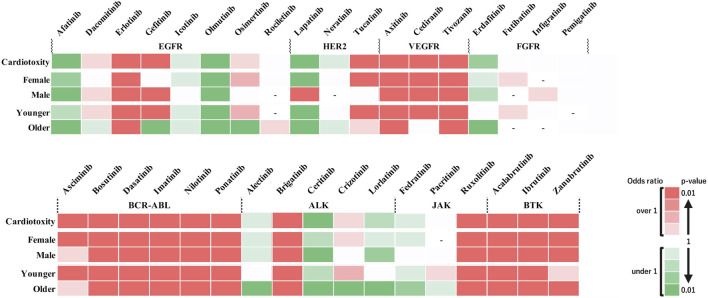
Risk assessment of adverse events induced by tyrosine kinase inhibitors across different subgroups. The heat map illustrates the risk of cardiotoxic adverse events associated with various tyrosine kinase inhibitors (TKIs). The drugs are categorized by receptor type (EGFR, HER2, VEGFR, FGFR, BCR-ABL, ALK, JAK, and BTK) along the horizontal axis, and the patient subgroups (sex and age categories) are categorized along the vertical axis. Color intensity indicates the level of risk based on the reported odds ratio (ROR) and P-value. The two darkest shades of red denote a significant risk signal (ROR > 1, P < 0.01), suggesting a higher incidence of adverse events in the database. Green shades indicate a ROR < 1, and the two darkest shades of green indicate a ROR < 1 and P < 0.01, representing a sufficient number of cases but no detected signal. Lighter shades of green denote subgroups with insufficient numbers of cases for validation. White cells indicate reports with relatively few cases to confirm significance. Cells with a dashed line (‘-‘) indicate no reports of that subgroup in the database (n = 0). This figure provides a visual representation of the comparative safety profiles of TKIs in reported data.

Figure 2 shows the heatmap created for the 35 types of TKIs and cardiovascular impairments under investigation. In the analyses using all included data, significant signals were identified for the following 17 drugs: erlotinib, gefitinib, tucatinib, axitinib, cediranib, tivozanib, ascriminib, bosutinib, dasatinib, imatinib, nilotinib, ponatinib, brigatinib, ruxolitinib, acalabrutinib, ibrutinib, and zanubrutinib. The stratified analysis revealed that lapatinib exhibited significant signals only in males. Gefitinib demonstrated significant signals exclusively in the younger patients. We calculated the IC using Bayesian estimation to confirm the certainty of our analysis. The results showed that TKIs with positive signals (i.e., IC025 > 0) were similar to those analyzed by ROR (Supplementary Table S1).

**FIGURE 3:**
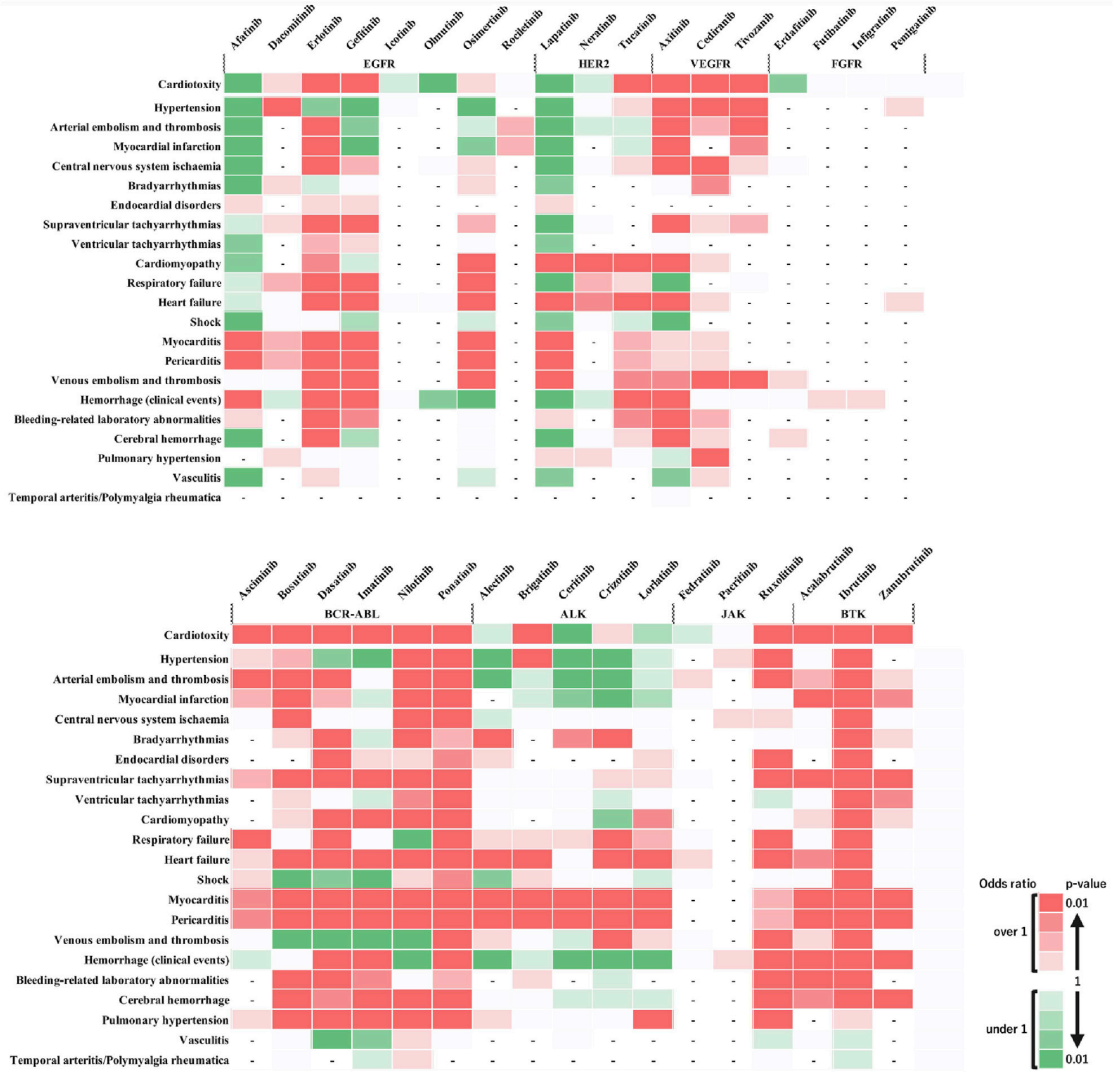
Comparative risk of adverse cardiovascular events induced by tyrosine kinase inhibitors. This heatmap provides a comparative analysis of the risk levels for various adverse cardiovascular events induced by different tyrosine kinase inhibitors (TKIs) along the horizontal axis against a range of cardiovascular adverse events listed on the vertical axis. The top row, labeled “Cardiotoxicity,” aggregates the overall cardiovascular risk associated with each drug. Two shades of dark red indicate a statistically significant signal (high risk), with the darkest shade representing the highest risk level. Green shades indicate an ROR < 1 or a P ≥ 0.5, with darker green representing P < 0.01, representing a substantial number of cases without a detected signal. Lighter shades of green represent subgroups with insufficient cases to validate the results. White cells denote reports with relatively few cases to assess significance. A dash (‘-‘) signifies no reports for the subgroup in the database (n = 0). This visual representation aids in understanding the relative safety profiles of TKIs in the context of cardiovascular events.

**FIGURE 4:**
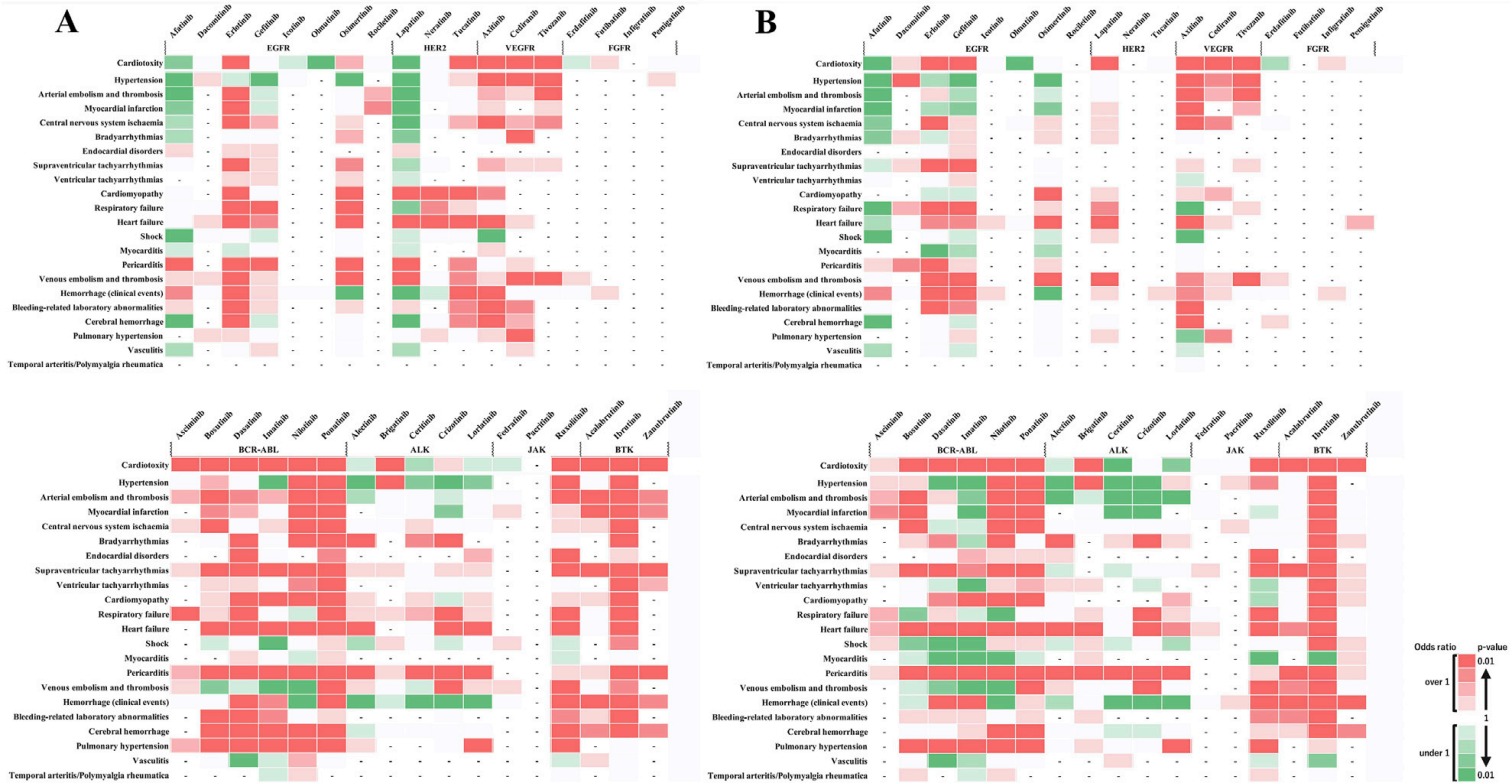
Sex-based subgroup analysis of adverse cardiovascular events induced by tyrosine kinase inhibitors. **(A)** represents the risk levels of various cardiovascular adverse events induced by tyrosine kinase inhibitors (TKIs) in female patients. **(B)** Illustrates the same for male patients. The color intensity in the heatmap indicates the level of risk based on the reported odds ratio (ROR) and P-value: Two shades of dark red indicate a statistically significant signal (high risk), with the darkest shade representing the highest risk level. Green shades indicate an ROR < 1 or a P ≥ 0.5, with darker green representing P < 0.01, representing a substantial number of cases without a detected signal. Lighter shades of green represent subgroups with insufficient cases to validate the results. White cells denote reports with relatively few cases to assess significance. A dash (‘-‘) signifies no reports for the subgroup in the database (n = 0). These visual representations help in understanding the relative safety profiles of TKIs in the context of cardiovascular events among different sexes.

Figure 3 shows the outcomes of each adverse event. These results revealed that when individual side effects were analyzed, drugs such as afatinib and lapatinib showed signals for specific side effects, although they did not show side effect signals in the analysis of cardiovascular toxicity as a whole (Figure 3). In stratified analysis by sex, HER2 inhibitors revealed significant signals for cardiomyopathy and heart failure in the female group but not in the male group (Figures 4A, B).

**FIGURE 5:**
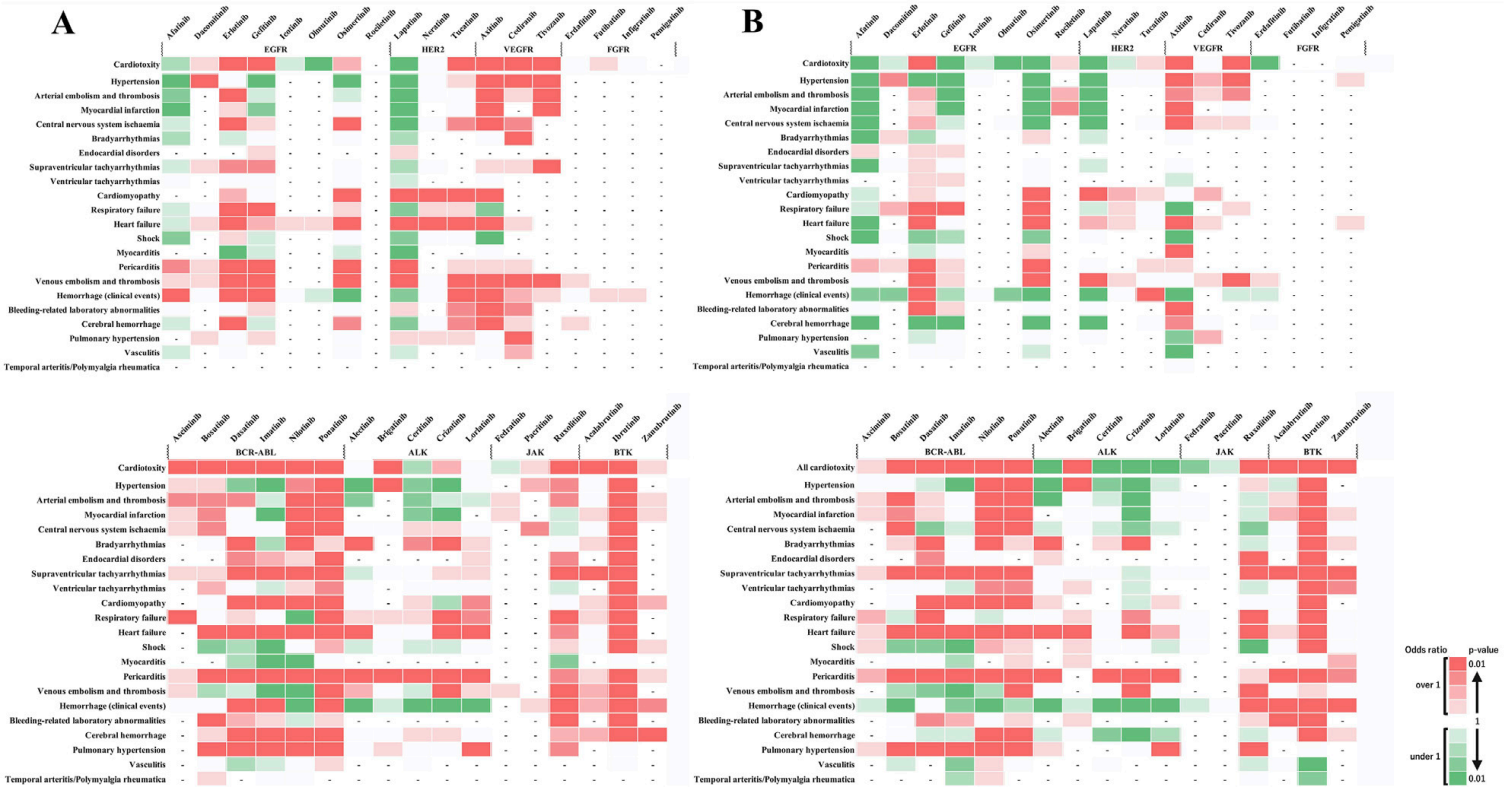
Age-based subgroup analysis of adverse cardiovascular events induced by tyrosine kinase inhibitors. **(A)** depicts the risk levels of various cardiovascular adverse events induced by tyrosine kinase inhibitors (TKIs) in younger patients. **(B)** Illustrates the same for older patients. The color intensity in the heatmap indicates the level of risk based on the reported odds ratio (ROR) and P-value: Two shades of dark red indicate a statistically significant signal (high risk), with the darkest shade representing the highest risk level. Green shades show an ROR < 1 or a P ≥ 0.5, with darker green representing P < 0.01, indicating a substantial number of cases without a detected signal. Lighter shades of green represent subgroups with insufficient cases to validate the results. White cells denote reports with relatively few cases to assess significance. A dash (‘-‘) signifies no reports for the subgroup in the database (n = 0). These visual representations help in understanding the relative safety profiles of TKIs in the context of cardiovascular events among different age groups.

Age-stratified analysis showed significant signals in the younger patients group for endocardial disorders with ponatinib (BCR-ABL) and pericarditis with brigatinib (ALK), with no reports in the older patients group (Figures 5A, B). No age-related differences were observed in other adverse events, indicating a potential risk of these adverse events in the older patients group. Conversely, significant signals were observed in the older patients group for bleeding-related laboratory abnormalities with acalabrutinib (BTK) and supraventricular and ventricular tachyarrhythmias with zanubrutinib (BTK), with no reports in the younger patients group (Figures 5A, B). The absence of age-related differences in other adverse events suggests a similar risk pattern across age groups.

## 4 Discussion

This study focused on elucidating the risk profile of cardiovascular impairment associated with TKIs based on a pharmacovigilance analysis using the VigiBase database. Our findings revealed that drugs classified as VEGFR TKIs, BCR-ABL TKIs, and BTK inhibitors exhibited similar potential risks for cardiotoxicity, in agreement with previous study results (Sayegh et al., 2023). Specifically for VEGFR TKIs, the results of axitinib regarding hypertension and bleeding align with those from previous reports; cediranib aligns with prior findings on hypertension but not bleeding in this study (Abdel-Rahman and Fouad, 2014).

Drugs grouped as EGFR, HER2, and ALK TKIs exhibited varied risk profiles for defined cardiovascular impairments (Figure 2). When the relationship between drugs and cardiovascular side effects was comprehensively assessed, HER2-associated drugs showed significant signals for cardiomyopathy and heart failure, whereas ALK-associated drugs demonstrated significant signals for heart failure, myocarditis, and pericarditis (Figure 3), suggesting that class-specific side effect profiles exist. Therefore, conducting class-specific risk assessments for some adverse events would be important. Moreover, adverse reactions in which similar signals were observed in patients of different genders and age groups are considered to be adverse reactions that require caution across a wide range of patients. This study underscores the importance of predicting cardiovascular toxicity risks based on existing drug data, including those with less clinical experience or newly approved drugs, to prevent adverse effects.

Our study showed that agents categorized in the same class, such as EGFR, HER2, and ALK TKIs, have different cardiovascular toxicity profiles, suggesting the presence of off-target effects (Chitturi et al., 2022). A study by Murtuza et al. (2019) indicated that adverse events, including heart failure with osimertinib, bradycardia with alectinib, and cardiomyopathy with lorlatinib, are due to off-target effects, which could explain the adverse events identified in this study. Potential targets or mechanisms of action include the activation of the HER2 or RAS-MAPK pathways (Murtuza et al., 2019). However, the scarcity of reports for drug groups, including FGFR-TKIs and JAK inhibitors, precluded their rendered evaluation in this study. Therefore, further data collection is necessary for reassessment.

To estimate the detailed side effect profile of drugs for which information is lacking, extrapolating the side effect profiles of drugs with established signals may be possible. For example, only a few reports of side effects for the JAK inhibitors, such as fedratinib or pacritinib, are available, and signal detection has not been sufficient (Figure 3), although side effect risk management similar to that for ruxolitinib may be useful. A sex difference was observed in the risk assessment of lapatinib; however, adverse events, such as heart failure, venous embolism, and thrombosis, showing significant signals were identified in both sexes. The lower number of male reports across all adverse events suggests the need for further verification with increased data accumulation among males. Currently, the sex-based differences in adverse events are inconclusive.

In previous reports consistent with this data, age-related risk differences associated with gefitinib have been revealed to show a significant risk of cardiovascular impairment in younger individuals. This finding aligns with the results of a previous report, suggesting a unique risk profile for the younger population (Kytö et al., 2013). Moreover, different signals for cerebral hemorrhage were observed with erlotinib and osimertinib between the younger and older groups, highlighting the necessity of age-specific risk management. The risk of hypertension was significant for drugs classified as BCR-ABL, including nilotinib and ponatinib, indicating the need for caution in patients with a history of hypertension. In summary, our findings suggest that cardiovascular impairments due to TKIs can be broadly categorized into the following two types: those shared by drugs classified based on their target molecules, such as VEGFR TKIs, BCR-ABL TKIs, and BTK inhibitors, and those that differ among drugs classified as EGFR, HER2, and ALK TKIs due to off-target effects.

The primary limitation of this study was the nature of the pharmacovigilance system database, which was based on spontaneous adverse drug reaction (ADR) notifications. ADRs are generally underreported, and the completeness of information varies across cases and countries (Baldo and De Paoli, 2014). Various factors, including the time on the market (new or old drug), the tendency to report only severe adverse events, and selective reporting of some drugs, could influence the reliability of detecting disproportionality signals (Arora et al., 2017; Bate and Evans, 2009). Moreover, the potential influence of concomitant medications on the outcomes was not assessed in this study. Concomitant drugs can have a significant impact on the occurrence and severity of adverse events, and their exclusion may introduce bias into the results. Therefore, further research using adverse event reporting systems such as VigiBase and identifiable patient information will enable a more detailed investigation of risk factors for adverse events.

Despite these limitations, this study is the first to investigate the potential risk of cardiovascular impairment associated with TKIs through disproportionality analysis using VigiBase data. While previous research has focused on the cardiovascular toxicity of specific TKIs, this study comprehensively investigated the cardiotoxic risk of TKIs, providing insights into the characteristics of drug classes. Therefore, this analysis is essential for understanding specific risk factors and devising individualized treatment plans.

## Data Availability

The datasets presented in this article are not readily available because the dataset was obtained from the WHO for a fee. Requests to access the datasets should be directed to https://who-umc.org/vigibase/vigibase-services/

## Abbreviations

TKIs, Tyrosine kinase inhibitors; TK, Tyrosine kinase; ATP, Adenosine triphosphate; ROR, Reported odds ratio; ADR, Adverse drug reaction; CIs, confidence intervals; ICIs, immune checkpoint inhibitors; FAERS, Food and Drug Administration Adverse Event Reporting System; UMC, Uppsala Monitoring Centre; IC, information component.
